# Antimicrobial resistance from a one health perspective in Cameroon: a systematic review and meta-analysis

**DOI:** 10.1186/s12889-019-7450-5

**Published:** 2019-08-19

**Authors:** Mohamed Moctar Mouliom Mouiche, Frédéric Moffo, Jane-Francis Tatah Kihla Akoachere, Ndode Herman Okah-Nnane, Nabilah Pemi Mapiefou, Valantine Ngum Ndze, Abel Wade, Félicité Flore Djuikwo-Teukeng, Dorine Godelive Tseuko Toghoua, Henri René Zambou, Jean Marc Kameni Feussom, Matthew LeBreton, Julius Awah-Ndukum

**Affiliations:** 1 Department of Pharmacy, Pharmacology and Toxicology, School of Veterinary Medicine and Sciences, University of Ngaoundéré, Ngaoundéré, Cameroon; 2 MOSAIC, Yaoundé, Cameroon; 3 Laboratory of Animal Physiology and Health, Department of Zootechnics, Faculty of Agronomy and Agricultural Sciences, University of Dschang, Dschang, Cameroon; 4 Department of Microbiology and Parasitology, Faculty of Science, University of Buea, Buea, Cameroon; 5 Institute of Agricultural Research for Development, Veterinary Research Laboratory, Wakwa Regional Center, Ngaoundéré, Cameroon; 6 Johns Hopkins Cameroon Program, Yaoundé, Cameroon; 7 National Veterinary Laboratory (LANAVET), Yaounde, Cameroon; 8 Faculty of Heath Science, Université des Montagnes, Bangangté, Cameroon; 9 National Public Health Laboratory (LNSP), Yaounde, Cameroon; 10 Epidemiology-Public Health-Veterinary Association (ESPV), Yaounde, Cameroon; 11 Cameroon Epidemiological Network for Animal Diseases (RESCAM), Ministry of Livestock, Fisheries and Animal Industries (MINEPIA), Yaoundé, Cameroon; 12 College of Technology, University of Bamenda, Bambili, Cameroon

**Keywords:** Antimicrobial resistance, Bacteria, One health, Human, Animal, Environment, Systematic review, Meta-analysis, Cameroon

## Abstract

**Background:**

Antimicrobial resistance (AMR) is widely acknowledged as a global health problem, yet in many parts of the world its magnitude is not well elucidated. A baseline assessment of the AMR prevalence is a priority for implementation of laboratory-based AMR surveillance This review, focused on a One health approach, aimed at describing the current status of AMR in Cameroon.

**Methods:**

PubMed, Google Scholar and African Journals Online databases were searched for articles published in English and French in accordance with the PRISMA guidelines. Retrieval and screening of article was done using a structured search string with strict inclusion/exclusion criteria. Free-text and grey literature were obtained by contacting the authors directly. The pooled prevalence and 95% confidence intervals were calculated for each pathogen–antibiotic pairs using random-effects models.

**Result:**

Amongst 97 full-text articles reviewed, 66 met the eligibility criteria. The studies originated from the Centre (24; 36.4%), South-West (16; 24.2%), West (13; 19.7%), Littoral (9; 13.6%) and other (4; 6.1%) regions of Cameroon. These studies reported AMR in human (45; 68.2%), animals (9; 13.6%) and the environment (12; 18.2%). Overall, 19 species of bacteria were tested against 48 antibiotics. These organisms were resistant to all classes of antibiotics and showed high levels of multidrug resistance. *Escherichia coli, Klebsiella pneumoniae* and *Staphylococcus spp* were reported in 23, 19 and 18 of the human studies and revealed multidrug resistance (MDR) rates of 47.1% [95% CI (37.3–57.2%)], 51.0% [95% CI (42.0–59.9)] and 45.2% [95% CI (38.0–54.7)], respectively. *Salmonella spp* was reported in 6 of the animal studies and showed a MDR rate of 46.2% [95% CI (39.2–53.5%)] while *Staphylococcus spp* in 8 of environment studies showed MDR rate of 67.1% [95% CI (55.2–77.2%)].

**Conclusion:**

This review shows that resistance to commonly prescribed antibiotics in Cameroon is high. The findings emphasize the urgent need to address gaps in the standardization of AMR diagnostics, reporting and use of available information to optimize treatment guidelines for the arsenal of antibiotics. Effective AMR surveillance through continued data sharing, large-scale collaboration, and coordination of all stakeholders is essential to understand and manage the AMR national burden.

## Background

The necessity to improve human health, animal health and agricultural productivity in low- and middle-income countries has led to the extensive use of antimicrobials without respecting therapy guidelines. However, farmers’ ignorance of the hazards related to antibiotic therapy and the widespread use of antimicrobials in food-producing animals has escalated the emergence of antimicrobial resistance (AMR) [1]. Thus, the worldwide public health concerns with respect to the antibiotics used in food-producing animals is due to the fact that they are closely related to those used in human medicine and select for resistance. Cross-species transmission of resistant bacteria or resistance genetic elements from animals or environment to humans has been reported [2, 3]. The World Health Organization (WHO) global report on AMR indicates that resistance of common bacteria has reached alarming levels in many parts of the world with high level resistance of *Escherichia coli* and *Klebsiella spp* to third-generation cephalosporin’s and carbapenems of up to 54% [4, 5]. High resistance rates have been described in bacteria isolated from food-producing animals to major antimicrobials used in human medicine [6–8]. In addition, high resistance rates in bacteria isolated from vegetables and environment have been reported [9].

Largescale and worldwide surveillance is critical to global AMR response. According to a WHO report, Africa has the largest gaps in data on AMR [5, 10]. A desktop analysis of the implementation of WHO’s Policy Package to combat AMR in the WHO African region revealed that two countries -Ethiopia and South-Africa (4.3%), have national AMR plans in place while 7(14.9%) other countries have only infection prevention and control policies. Four of these countries are from Eastern Africa (Tanzania, Zimbabwe, Ethiopia, and Kenya), two from southern Africa (Lesotho and South Africa) and one West Africa (Ghana). Furthermore, no African country has a national surveillance system that routinely generates representative, robust data on antimicrobial use and resistance [11].

There is dearth of information on AMR in livestock and agricultural sectors in Africa. Moreover, major gaps exist in the surveillance and sharing of data on AMR emergence among food-borne bacteria and the potential impact on animal and human health [12]. Due to resource constraints, the choice of antibiotic in most parts of Africa including Cameroon is usually not based on knowledge of bacterial-susceptibility. Lack of reliable and quantitative data challenges local and regional treatment guidelines and highlights the need for sustainable efforts by stakeholders towards coordination and harmonization of competences to assess and monitor AMR emergence.

AMR is suspected when there is therapeutic failure evidenced by no convalescence. Limited diagnostic capacity coupled with a high burden of life-threatening bacterial infections has encouraged a pattern of largely empirical antibiotic prescription in human and veterinary medicine in Cameroon. Also, a small repertoire of generic antimicrobials which are often poor quality [13], sold in open markets with little or no regulations, quality control and inappropriate prescription practices [13, 14], are regularly used. Poor personal and environmental hygiene practices have favored the spread of drug resistant pathogens within the hospital environment, causing hospital-acquired infections [15, 16]. The use of antimicrobials as growth promoters in agriculture has been associated with resistant food borne pathogens and relatively risky to human, animal and environmental health [17]. It is based on this context that this review was done to analyze the available information on AMR emergence in humans, animal and environment as key to effective interventions in Cameroon.

## Methods

### Search strategy

**Fig. 1:**
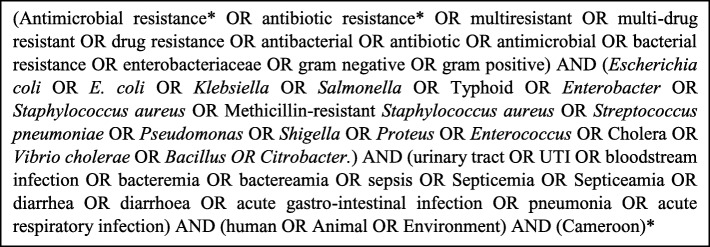
Search terms used to identify relevant literature from PubMed, Google Scholar and African Journals Online databases. *These search terms were translated to search articles written in French

The systematic review was performed in accordance with PRISMA (Preferred Reporting Items for Systematic Reviews and Meta-Analysis) guidelines [18]. PubMed, Google Scholar and African Journals Online databases were used to search articles published in English and French on AMR in Cameroon. Free-text and grey literature were obtained by contacting authors directly. No limit on publication dates was set. Literature search started on March 22, 2018, with an update on December 30, 2018. Reference list of relevant articles were checked for additional titles for inclusion in the review. The Boolean search strategy [19] with search terms pertaining to antibiotic resistance, bacteria of interest in relevant study conducted in Cameroon in human and animal health, and the environment was adopted (Fig. 1). These bacteria are important causes of infections in both animal and human and were enrolled in countries’ AMR surveillance by the Global Antimicrobial Resistance Surveillance System (GLASS) [20].

### Inclusion and exclusion criteria

Full-text articles on the prevalence of antibiotic resistance among clinical pathogenic bacteria isolated from humans (inpatients and outpatients, healthy volunteers), animals and animal-products (avian, cattle, swine, fish, meat, milk, eggs) and environment (water, hospital surfaces and health care tools, vegetables sold in the market, drainages) in Cameroon were used for the review. Publications were independently reviewed by three authors (FM, MMMM and NPM) to determine eligibility. Disagreements were resolved by consensus or third-party consultation (JAN and JFTKA) when consensus could not be achieved. Publications that described human subjects or livestock population studied or type of environmental samples, bacteria isolated, and specific laboratory methods, antimicrobial sensitivity patterns, interpretation of minimum inhibitory concentration breakpoints and diameter of the zone of inhibition of the antibiotics tested were considered and included in the study. As concerns studies related to human, both adult and pediatric patient populations were included. Studies on tuberculosis, non-bacterial pathogens and outbreak disease were not included. Studies reporting aggregated data such as studies with the methodology aggregating resistance rates in a large category such as ‘Gram-negative organisms’, Gram-positive organisms or ‘Enterobacteriaceae’ were excluded. As concern animal and environmental studies, articles on prevalence related to aggregate resistance rates and without information on total bacteria isolates were excluded from the review. Also, articles identified through the literature search that reported AMR in human, animal and environment but that did not report prevalence data were not included in the meta-analysis.

### Data extraction

Data was extracted from individual study using a form and database developed for the purposes of this review using Microsoft Excel 2013. The data extraction was independently done by three co-authors (FM, MMMM and NPM). When there was confrontation of data set, third-party (JAN and JFTKA) consultation was sort for validation. Articles that met the inclusion criteria and reported prevalence data for AMR in bacteria of interest were included in the meta-analysis. Information extracted included article information (first author, year of publication, duration of study and location), and study design (samples size, cross-sectional design or longitudinal study). Specific information on human considered: category of patients (in- or out-patients), type of samples used (pus, blood, throat swabs, stool, nasal swabs, urine, vaginal or wound swabs). In animal: species, number of farms or animals used in analysis, sample type (faeces, meat, milk, blood), sampling point (farm, slaughter house, or retail market) while for the environment, information of interest as water, vegetables, drains, hospital surfaces and medical tools were considered. Antibiotic panels, laboratory procedure, proportion of bacteria investigated and prevalence of antibiotic-resistant bacteria were taken into consideration for the human, animal and environment articles. The following bacteria were included in the search: *Escherichia coli*, *Klebsiella spp, Salmonella spp*, *Staphylococcus spp, Streptococcus spp, Shigella spp, Proteus spp, Enterococcus spp, Enterobacter spp, Citrobacter spp, Pseudomonas spp, Vibrio spp* and *Bacillus spp.*

### Data analysis

The point estimate prevalence and 95% confidence interval (CI) of each pathogen–antimicrobial pair in human, animal and environment was pooled using a random effects model. Random-effects meta-analysis was also used to calculate an overall proportion of pathogen–multidrug resistance pair. If not defined by the study, resistance to two or more antibiotics, frequently used in the primary reports, was considered as multidrug resistance (MDR) [2].

Quality assessment of articles was done using the modified critical appraisal tool developed by Munn et al. [21]. Heterogeneity across the studies was assessed using the Cochrane Q statistics (significant at *p* < 0.10) and was quantified with the I^2^ statistic [19, 22]. Sensitive analysis was performed to evaluate the influence of individual studies on the final effect. The Begg rank correlation [23] and Egger regression asymmetry test [24] were used to examine publication bias. If publication bias was confirmed, a trim-and-fill method developed by Duval and Tweedie [25] was implemented to adjust for the bias. The funnel plot was replicated with their *“*missing*”* counterparts around the adjusted summary estimate. If after detailed investigation, there was no obvious cause for the heterogeneity; data was analyzed with a more conservative statistical method that accounted for it. The random-effects meta-analysis attempts to account for the distribution of effects and provided more conservative estimate of the effect [22, 26]. Subgroup analysis was performed according to the region of the country.

Meta-analysis was done separately for data extracted from the human, animal and environmental articles. *P*-value of 0.05 was considered statistically significant, except in the test of heterogeneity. Data were analyzed using Comprehensive Meta-Analysis Software (Biostat, Inc., New Jersey) Version 3.0 for Windows. In human, point estimates resistance was calculated where if at least four studies reported on the specific bacterium-antibiotic combination, while for animals and environment, it was estimated where three studies reported the same combination (specific bacterium-antibiotic). Overall, MDR prevalence was pooled when five human studies, four animal or environment studies reported combination of specific bacteria resistance to more than two antibiotics.

## Results

**Fig. 2:**
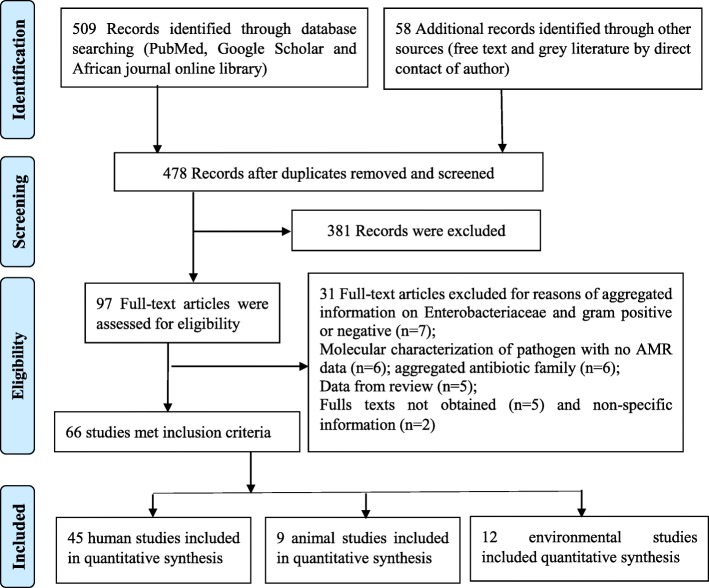
Prisma Flow-chart illustrating the study selection process on antimicrobial resistance in Cameroon

**Fig. 3:**
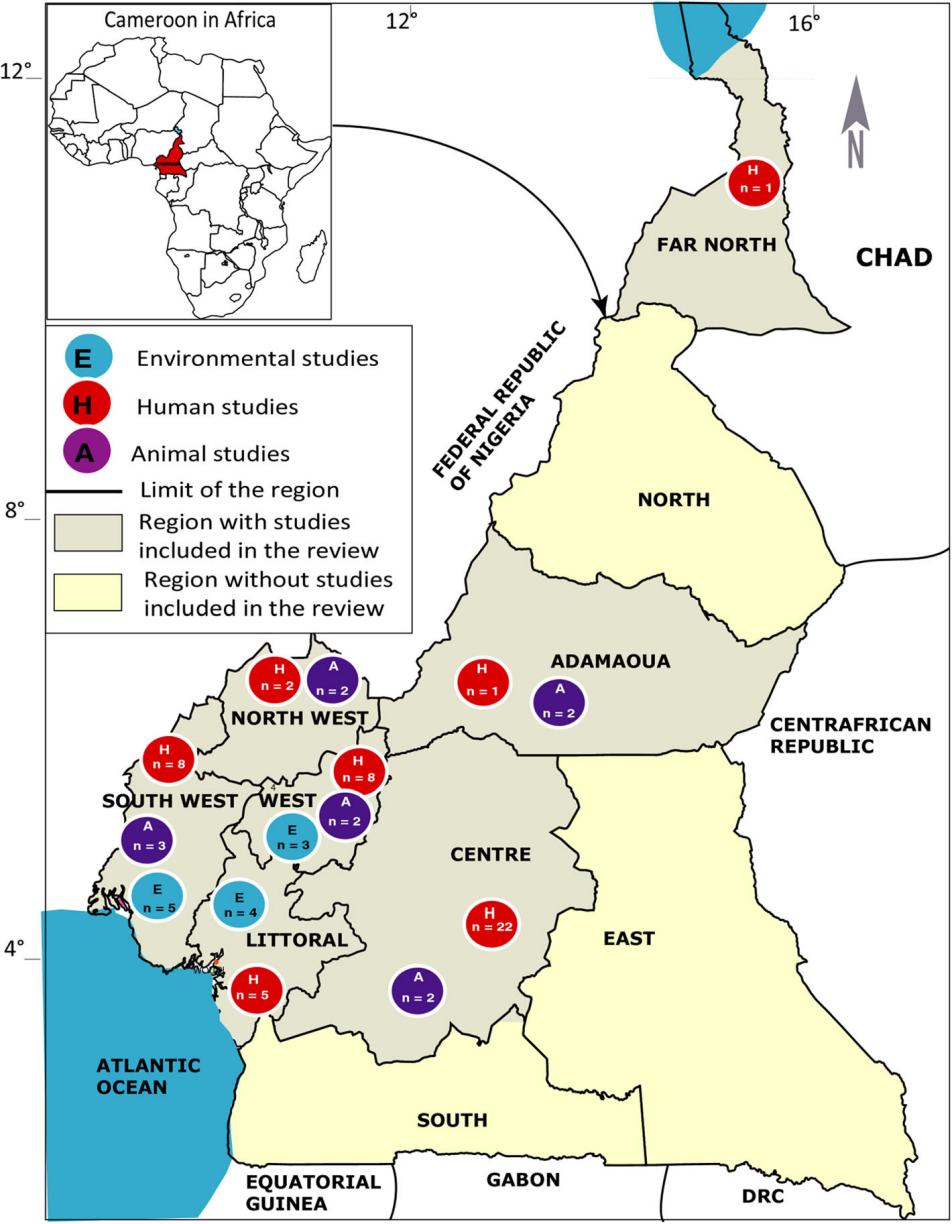
Map of the Cameroon showing study sites and the number of articles used in the review

The initial search strategy of the online databases in this study identified a total of 509 citations. Additional 58 records were identified by contacting experts in the field of antibiotic use and resistance (12), searching reference lists of included studies (44) and local database (2). A total of 89 duplicates were removed and 478 records were screened for eligibility based on review of title and content of the abstracts. Overall, 381 records were excluded for non-relevance to the research objectives while 97 full-text articles were assessed for eligibility with 66 records meeting the inclusion criteria for the study (Fig. 2). Of these 66 studies included in the review, 52 were rated as good quality (low risk of bias) and 14 were of moderate quality (medium risk of bias). A total of 45 studies reported on the outcome of antibiotic resistance in humans [15, 27–69], 9 reported on the outcome of antibiotic resistance in animals [70–78] and 12 on the environment [9, 15, 16, 57, 62, 64, 75, 79–83] (Table 1). Five studies reported outcome of antibiotic resistance in both human and environment while one study reported on the outcome of antibiotic resistance for animals, humans and environment. The largest number of human studies originated from the Centre region (46.7%) followed by the West (17.8%), South-West (15.6%), Littoral (11.1%) and other (North-West, Adamawa, Far-North) (4.4%) regions of the country (Fig. 3). These sources were journal articles (100%), published in English (88.9%) with focus on both sex (95.6%) and 4.4% strictly on women. These studies investigated Urinary Tract infections (UTIs) (29; 64.4%), Bloodstream infections (BSIs) (17; 37.8%), Gastro-intestinal infections (GIIs) (15; 33.3%), and Respiratory tract infections (RIs) (14; 31.1%). Overall, 25 types of samples were reported in the various studies, the most abundant being urine samples (26; 57.8%), followed by vaginal swabs (14; 31.1%), stool samples (14; 31.1%), pus (12; 26.7%), blood (12; 26.7%), wounds swabs (10; 22.2%) and others: skin (6; 13.3%), nasal swabs (4; 8.9%), Urinary catheter (4; 8.9%), pleural fluid (4; 8.9%), seminal fluid (3; 6.7%), bone fragment (3; 6.7%), septum (3; 6.7%), sinusitis (3; 6.7%), fingerprinting (3; 6.7%), throat swabs (2; 4.4%), eye discharge (2; 4.4%), ear discharge (2; 4.4%), urethral smear (2; 4.4%), corpus and antral biopsy (1; 2.2%), surgical site (1; 2.2%), cerebrospinal fluid (1; 2.2%), ostitis (1; 2.2%) and bronchitis (1; 2.2%).

**Table 1:** Distribution and characteristics of studies used in the review

	Number of human studies (*n* = 45)	Number of animal studies (*n* = 9)	Number of environmental studies (*n* = 12)
Region			
Adamawa	1 (2.2%)	2 (22.2%)	
Centre	21 (46.7%)	2 (22.2%)	
Far-north	1 (2.2%)		
Littoral	5 (11.1%)		4 (33.3%)
North-west	2 (4.4%)		
South-west	7 (15.6%)	3 (33.3%)	5 (41.7%)
West	8 (17.7%)	2 (22.2%)	3 (25%)
Type of material			
Journal article	45 (100%)	7 (77.8%)	12 (100%)
Dissertation (Unpublished)		2 (22.2%)	
Language			
English	40 (88.9%)	5 (55.6%)	12 (100%)
French	5 (11.1%)	4 (44.4%)	
Study design			
Cross sectional	42 (93.3%)	9 (100%)	12 (100%)
Longitudinal	2 (6.7%)		
Animal and human population study			
Beef cattle		2 (22.2%)	
Poultry: broilers & eggs layers		5 (55.6%)	
Swine		1 (11.1%)	
Shrimps		1 (11.1%)	
Healthy adults	5 (11.1%)		
Patients or cases	36 (80%)		
Health care personnel	4 (8.9%)		
Common infection			
Urinary tract infection	29 (64.4%)		
Bloodstream infection	17 (37.8%)		
Gastro-intestinal infection	15 (33.3%)		
Respiratory infection	14 (31.1%)		
Skin problems	6 (13.3%)		
Eye and ear discharge	5 (11.1%)		
Samples studied			
Faecal/cloacal swabs/caecum		7 (77.8%)	
Meat or carcass		1 (11.1%)	
Shrimps		1 (11.1%)	
Stool sample	14 (31.1%)		
Urine	26 (57.8%)		
Vaginal swabs	14 (31.1%)		
Blood	12 (26.7%)		
Pus	12 (22.7%)		
Wounds swabs	10 (22.2%)		
Skin	6 (13.3%)		
Nasal swabs	4 (8.9%)		
Medical tools and devices			6 (50%)
Water			3 (25%)
uncooked vegetables			1 (8.3%)
Money (notes and coins)			1 (8.3%)
Abattoirs drains			1 (8.3%)
Bacteria isolates studied			
*Escherichia coli*	23 (51.1%)	3 (33.3%)	3 (25%)
*Salmonella spp.*	1 (2.1%)	6 (66.7%)	1 (8.3%)
*Klebsiella spp*	19 (42.2%)		3 (25%)
*Enterococcus spp*	7 (15.6%)	1 (11.1%)	2 (16.7%)
*Proteus spp*	6 (13.3%)	2 (22.2%)	2 (16.7%)
*Citrobacter spp*	4 (8.9%)		
*Staphylococcus spp.*	18 (40%)	1 (11.1%)	8 (66.7%)
*Streptococcus spp*	6 (13.3%)		1 (8.3%)
*Pseudomonas aeruginosa*	11 (24.4%)		4 (33.3%)
*Bacillus spp*	2 (4.4%)		6 (50%)
*Shigella spp*	2 (4.4%)		3 (25%)
*Vibrio cholerae*	1 (2.1%)	1 (11.1%)	3 (25%)

Nineteen types of pathogens were isolated from samples with the most frequently encountered isolates being *Escherichia coli* (51.1%) followed by *Klebsiella spp* (42.2%), *Staphylococcus spp* (40%), *Pseudomonas aeruginosa* (24.4%), *Enterobacter spp* (17.8%) and *Enterococcus spp* (15.6%). These isolates were tested against 48 antimicrobial agents belonging to 14 families. The studies were conducted in urban settings (34/45; 75.5%), semi-urban (8/45; 17.8%) and rural areas (3/45; 6.7%). The majority of patients were hospitalized (30/45; 66.7%) while 19 (42.2%) were outpatient, 10 (22.2%) members of the community, 6 (13.3%) medical staff and 2 (4.4%) non-medical staff. These were cross sectional prospective studies (93.30%) and longitudinal studies (6.7%). With regards to the type of microbiology laboratory used, the studies were carried out in teaching hospitals partnering with an associated laboratory (22/45; 48.9%), routine clinical laboratories (19/45; 42.2%) as well as research laboratories (4/45; 8.9%). The disk diffusion test (37/45; 82.2%) was predominantly used for antibiotic susceptibility testing followed by dilution test (4/45; 8.9%) and Etest (2/45; 4.4%). Some studies used both diffusion and dilution test (2/45; 4.4%) and diffusion and Etest (1/45; 2.2%). The following microbiological standards were used as references: the Clinical Laboratory Standard Institute (20/45, 44.4%), the French Society of Microbiology (16/45; 35.6%), European Committee on Antimicrobial Susceptibility Testing (1/45; 2.2%), American Type Culture Collection (1/45; 2.2%), and both French Society of Microbiology and European Committee on Antimicrobial Susceptibility Testing (3/45; 6.7%). Four (8.9%) studies did not clearly define the susceptibility laboratory references used.

Animal studies originated from four regions: South-West (33.3%), Adamawa (22.2%), Centre (22.2%), and West (22.2%) while environmental studies were from South-West (41.7%), Littoral (33.3%) and West (25%) regions (Fig. 3). Seven animal’s studies were journal articles published in English and two Doctor of Veterinary Medicine dissertations written in French. A total of 12 environmental studies were journal articles published in English. Animal and environmental studies were cross-sectional designs. Poultry (5/9; 55.6%) was the most studied animal population, followed by beef cattle (2/9; 22.2%). Faecal/cloacal swabs/caecum (7/9; 77.8%) were the most harvested samples in animal studies while in environmental studies, swabs of hospital surfaces and medical devices (50%) and water samples (25%) were the most investigated. Results reported *Salmonella spp* (66.6%) and *Escherichia coli* (33.3%) as the most isolated organisms in animal studies and *Staphylococcus spp* (66.7%), *Bacillus spp* (50%) and *Pseudomonas aeruginosa* (33.3%) in environmental studies. For the animal studies, 7 articles made use of disk diffusion tests while 2 used both diffusion and dilution tests. The Clinical Laboratory Standard Institute standards were used as reference in 5(55.6%) studies while the rest used the French Society of Microbiology standards (4/9; 44.4%). All environmental studies made use of disk diffusion tests, but microbiological reference standards of the Clinical Laboratory Standard Institute were used by 63.6%) (7/11) and the French Society of Microbiology by 36.4% (4/11).

### Antibiotic resistance rates in human studies

**Fig. 4:**
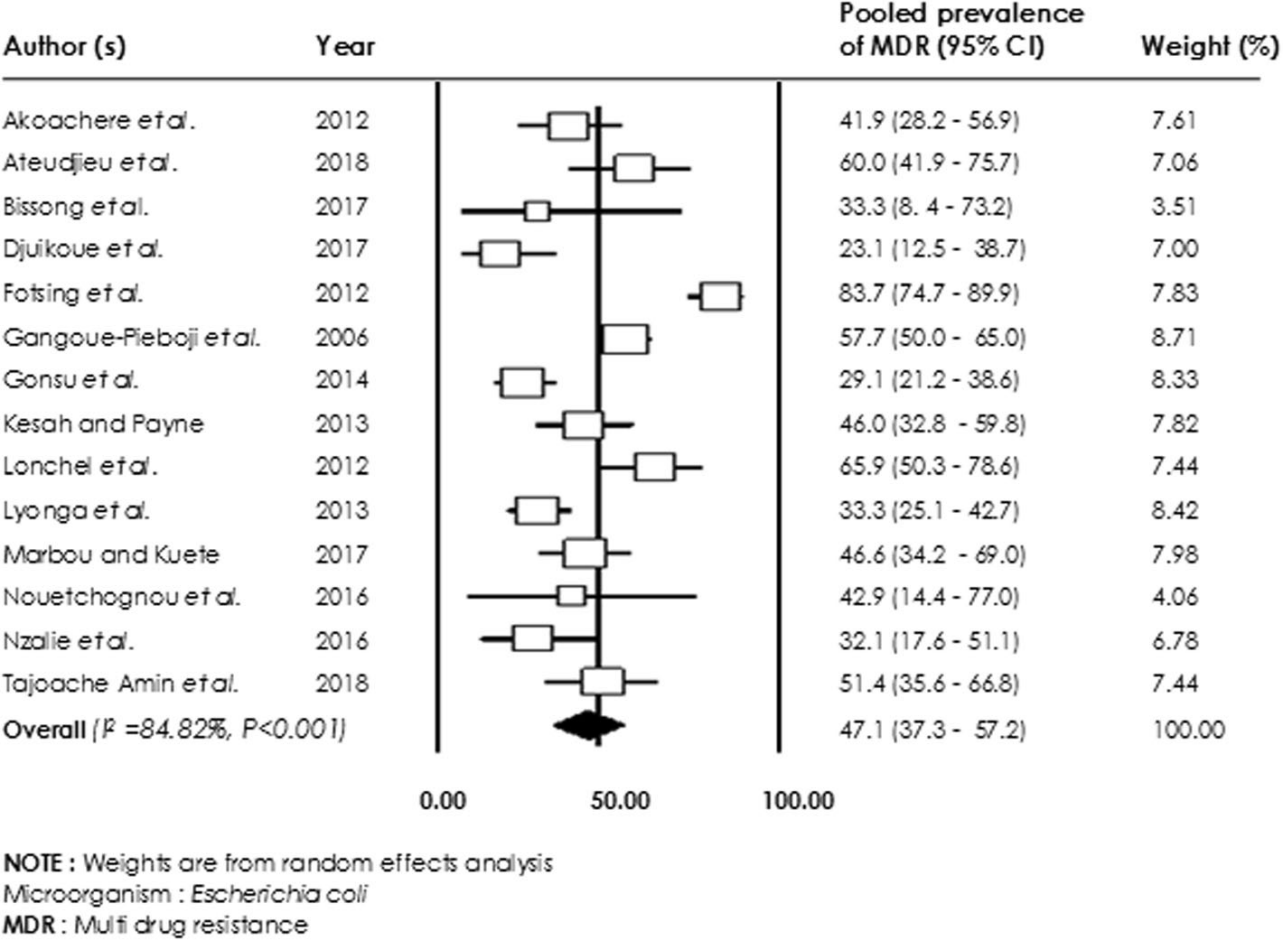
Forest plot of pooled prevalence of *Escherichia coli*-multidrug resistance in human

Of the 45 studies reporting antibiotic resistance in human, 34 were included in the meta-analysis. Bacterial resistance rates were carried out without distinguishing between patient and healthy volunteers (Table 2). Generally, a higher level of resistance of *E. coli*, *Klebsiella spp*, *Staphylococcus spp*, *Pseudomonas aeruginosa*, *Enterobacter spp,* and *Proteus spp* to all classes of antibiotics was observed. Lower levels of *E. coli* resistance were observed for Gentamicin 36.5% [95% CI (23.7–51.5%)], Ceftriaxone 38.7% [95% CI (25.7–53.6%)] and Ciprofloxacin 39.1% [95% CI (28.1–51.2%)] compared with resistance to Tetracycline 71.9% [(95% CI (66.1–88.0%)], Amoxicillin 77.3% [95% CI (59.7–88.7%)] and Trimethoprim / Sulfamethoxazole 85.2% [95% CI (69.1–93.7%)]. Overall, an *E. coli* multidrug resistance rate of 47.1% [95% CI (37.3–57.2%); I^2^ = 84.82%; *p* < 0.001)] was observed (Fig. 4).

**Table 2:** Pooled prevalence rates of antibiotic resistance of bacteria based on a meta-analysis of human studies

Bacteria reported in the studies reviewed	Antimicrobial agent	Number of studies	Pooled prevalence of AMR (95% CI)
*Escherichia coli*	Beta-lactams		
	Amoxicillin	10	77.3 (59.7–88.7)
	Amoxicillin+ Clavulanic acid	8	63.3 (48.9–75.9)
	Ampicillin	4	65.6 (37.9–85.6)
	Ceftriaxone	7	38.7 (25.7–53.6)
	Aminoglycosides		
	Gentamicin	12	36.5 (23.7–51.5)
	Quinolones		
	Nalidixic Acid	7	53.5 (35.1–71.0)
	Ciprofloxacin	11	39.1 (28.1–51.2)
	Tetracyclines		
	Doxycycline	4	45.0 (37.0–53.4)
	Tetracycline	5	71.9 (66.1–88.0)
	Nitrofuranes		
	Nitrofurantoin	5	79.7 (66.1–88.0)
	Phenicols		
	Chloramphenicol	4	50.2 (27.0–73.3)
	Sulfonamides & Trimethoprim		
	Trimethoprim/Sulfamethoxazole	6	85.2 (69.1–93.7)
	Co-trimoxazole	4	48.8 (38.4–59. 3)
*Klebsiella spp*	Beta-lactams		
	Amoxicillin	6	94.7 (84,9–98.3)
	Ampicillin	4	76.1 (52.5–90.1)
	Ceftriaxone	5	46.0 (32.3–60.4)
	Aminoglycosides		
	Gentamicin	9	44.1 (23.5–67.0)
	Quinolones		
	Ciprofloxacin	8	26.7 (15.7–42.0)
	Phenicols		
	Chloramphenicol	6	60.0 (30.3–83.8)
	Sulfonamides & Trimethoprim		
	Trimethoprim/Sulfamethoxazole	4	78.3 (69.6–85.1)
	Co-trimoxazole	5	76.8 (47.5–92.7)
*Staphylococcus spp*	Beta-lactams		
	Amoxicillin	5	65.2 (51.8–76.5)
	Amoxicillin+Clavulanic acid	9	50.8 (36.4–65.1)
	Penicillin	9	95.8 (85.2–98.9)
	Ampicillin	5	60.9 (27.6–86.5)
	Oxacillin	5	92.0 (46.1–99.4)
	Ceftriaxone	8	56.7 (23.7–88.7)
	Ceftazidime	6	52.9 (23.4–80.5)
	Cefotaxime	5	91.1 (77.4–96.9)
	Aminoglycosides		
	Gentamicin	15	43.3 (23.7–65.3)
	Macrolides		
	Erythromycin	11	55.7 (47.7–63.4)
	Quinolones		
	Ciprofloxacin	9	42.9 (20.6–68.6)
	Sulfonamides & Trimethoprim		
	Trimethoprim/Sulfamethoxazole	5	81.5 (52.5–94.6)
	Co-trimoxazole	8	73.0 (47.5–89.0)
	Tetracyclines		
	Doxycycline	6	53.5 (34.2–71.8)
	Tetracycline	5	68.0 (59.3–75.5)
	Nitrofuranes		
	Nitrofurantoin	6	34.1 (22.3–48.2)
*Pseudomonas aeruginosa*	Monobactams		
	Aztreonam	5	59.0 (28.9–83.6)
	Beta-lactams		
	Ceftazidime	6	24.8 (15.8–37.0)
	Cefotaxime	4	78.6 (60.6–89.8)
	Aminoglycosides		
	Gentamicin	6	46.9 (38.2–55.8)
*Enterobacter spp*	Aminoglycosides		
	Gentamicin	5	30.6 (20.2–63.0)
	Quinolones		
	Ciprofloxacin	4	56.3 (36.6–61.7)
	Sulfonamides & Trimethoprim		
	Co-trimoxazole	4	83.2 (61.2–93.9)
*Proteus spp*	Beta-lactams		
	Amoxicillin	4	61.1 (40.2–78.5)
	Amoxicillin+ Clavulanic acid	4	49.8 (23.1–76.6)
	Aminoglycosides		
	Gentamicin	4	34.7 (26.3–44.2)

**Fig. 5:**
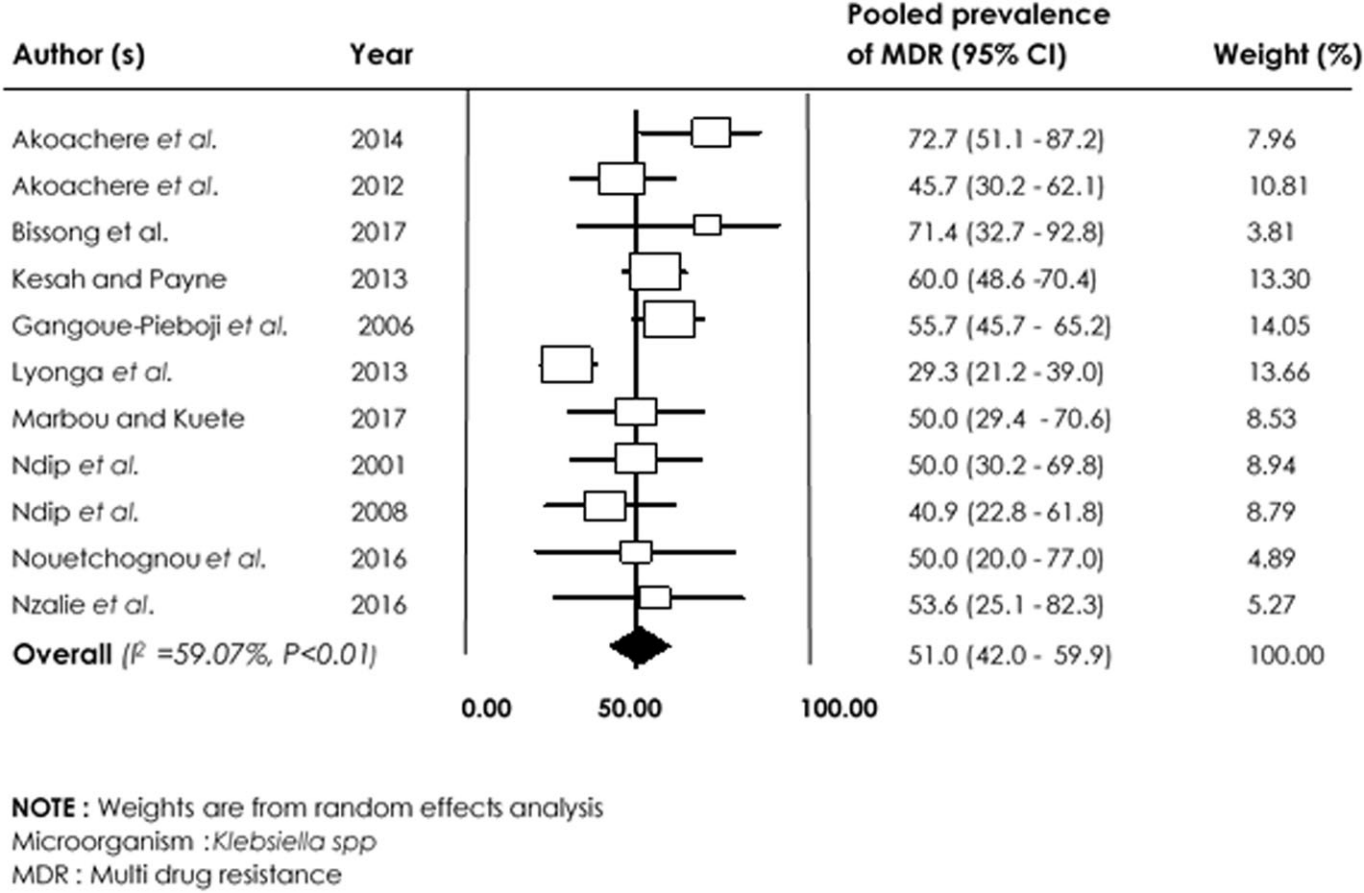
Forest plot of pooled prevalence of *Klebsiella spp*-multidrug resistance in human

With regards to *Klebsiella spp*, low resistance to Ciprofloxacin 26.7% [95% CI (15.7–42.0%)] and Gentamicin 30.6% [95% CI (20.2–63.0%)] was observed compared with Trimethoprim/Sulfamethoxazole 78.3% [95% CI (69.6–85.1%)] and Amoxicillin 94.7% [95% CI (84.9–98.3%)]. Overall, the prevalence of multidrug resistance in *Klebsiella spp* was 51.3% [95% CI (42.5–60.0); I^2^: 59.07%; *p* < 0.01] (Fig. 5).

Among *Staphylococcus spp* isolates*,* the lowest pooled resistance rates were in Nitrofurantoine 34.1% [95% CI (22.3–48.2%)] and Ciprofloxacin 42.9% [95% CI (20.6–68.6%)] compared with other commonly used agents such as Beta-lactams with high level of AMR (Table 2).

**Fig. 6:**
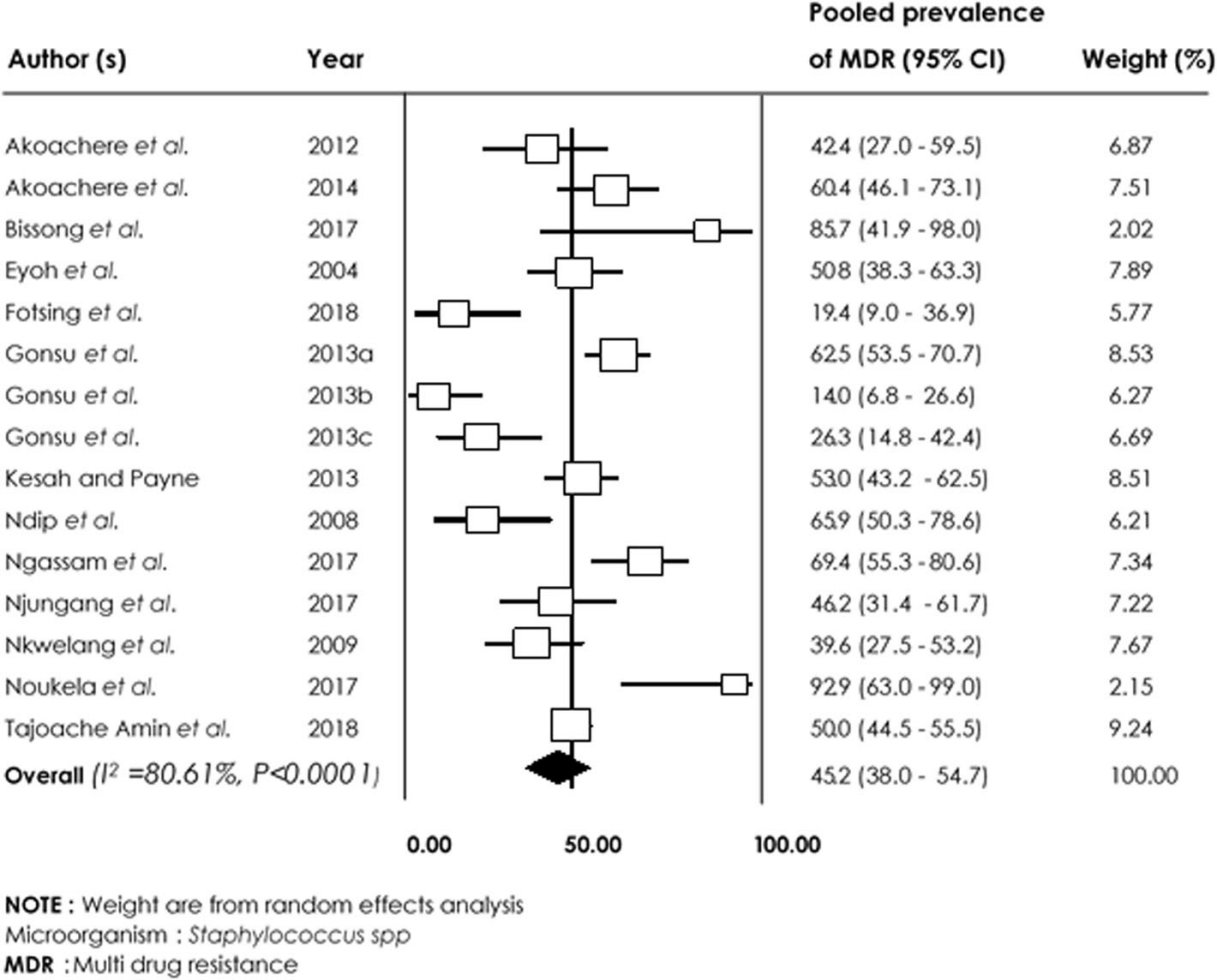
Forest plot of pooled prevalence of *Staphylococcus spp*-multidrug resistance in human

With regards to Beta-lactams, pooled AMR ranged from 52.9% [95% CI (23.4–80.5%)], to 91.1% [95% CI (77.4–96.9%)] for Ceftazidime and Cefotaxime, respectively. High resistance rates were also observed in Trimethoprim/Sulfamethoxazole 81.5% [95% CI (52.5–94.6%) and Penicillin 95.8% [95% CI (85.2–98.9%)]. Multidrug resistance rate of 45.2% [95% CI (38.0–54.7%; I^2^ = 80.61%; *p* < 0.0001)] was recorded in *Staphylococcus spp* (Fig. 6).

**Fig. 7:**
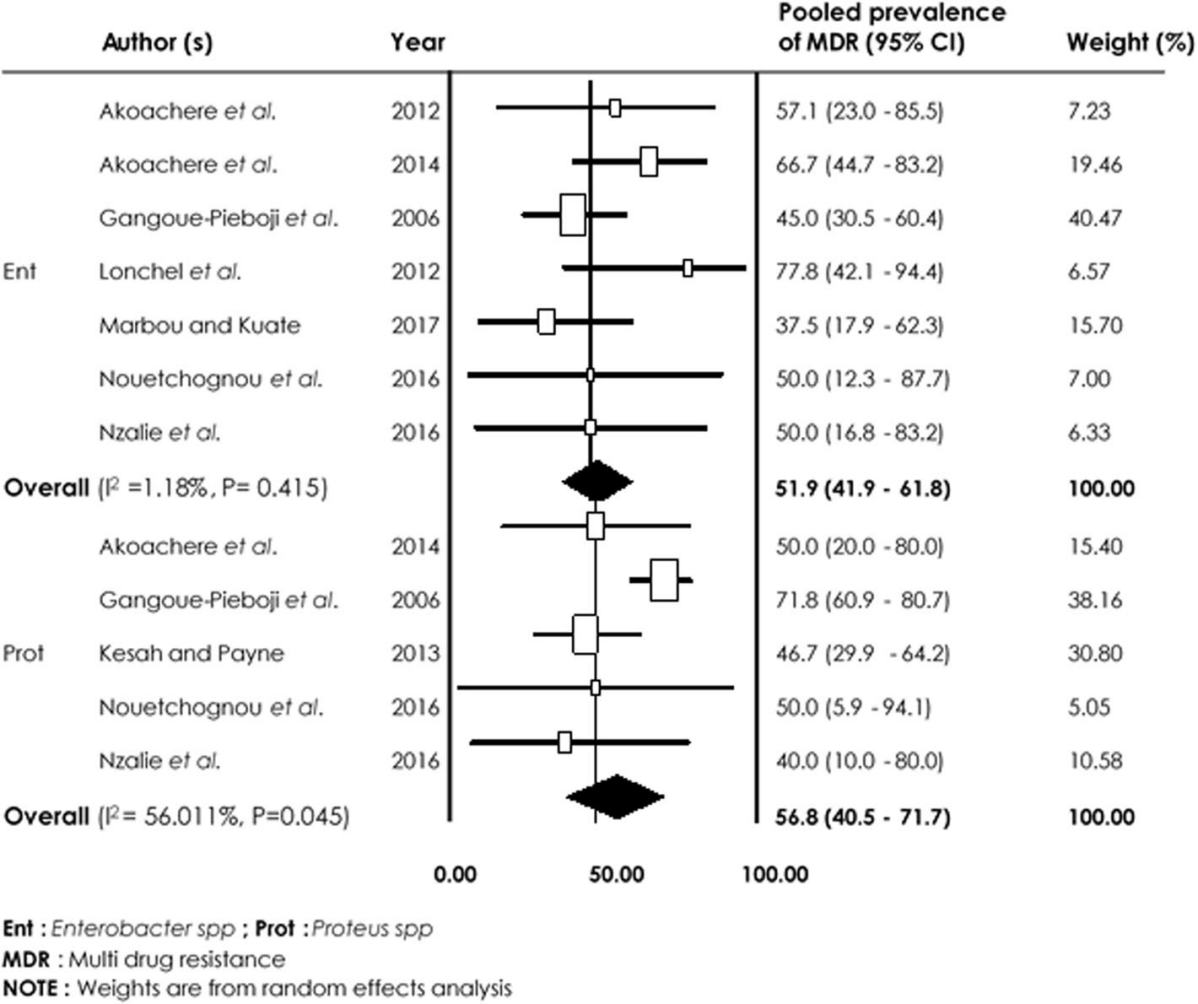
Forest plot of pooled prevalence of *Enterobacter spp and Proteus spp*-multidrug resistance in human

As concerned *Pseudomonas aeruginosa, Enterobacter spp,* and *Proteus spp,* the lowest pooled resistance rate was 24.8% [95% CI (15.8–37.0%)] to Ceftazidime and 30.6% [95% CI (20.2–63.0%)] to Gentamicin. MDR prevalence rates of 51.9% [95% CI (41.9–61.8%), I^2^ = 1.18%, *p* = 0.415] and 56.8% [95% CI (40.5–71.7%), I^2^ = 56.011%, *p* = 0.0045] were observed for *Enterobacter spp* and *Proteus spp*, respectively (Fig. 7).

Based on the region where the studies were reported (Table 3), the pooled estimate of MDR was moderate to high of *E. coli, Staphylococcus spp* and *Klebsiella spp* regularly isolated in hospital settings. However, there was no significant difference (*p* > 0.05) in multidrug resistance of respective pathogens across regions.

**Table 3:** Pooled prevalence of Multidrug Resistance of *E. coli*, *Klebsiella spp* and *Staphylococcus spp*- based on meta-analysis of human studies with respect to study area

Bacteria mostly reported in studies	Study Area (Region)	Number of studies	Pooled prevalence of AMR (95% CI)	*p* value
*E. coli*	Centre	6	35.9 (24.2–49.6)	0.25
	South-West	3	45.6 (35.6–56.0)	0.82
	North-West	3	60.9 (33.0–83.2)	0.33
	West	3	40.9 (28.1–55.0)	0.61
*Klebsiella spp*	Centre	4	45.8 (28.3–64.1)	0.65
	South-West	4	51.6 (38.5–64.5)	0.82
	North-West	2	52.6 (30.5–73.7)	0.64
	West	2	57.9 (47.7–67.4)	0.13
*Staphylococcus spp*	Centre	5	39.2 (23.2–57.8)	0.45
	South-West	2	62.2 (18.2–92.4)	0.20
	North-West	5	45.3 (36.2–54.7)	0.69
	West	4	56.6 (32.0–78.4)	0.45

Note: *P* values are from random effects analysis, AMR: Antimicrobial resistance

### Antibiotic resistance rates in animals

Out of 9 studies reporting antibiotic resistance in animals, 7 were included in meta-analysis. Resistance rates were pooled if at least three studies reported on a specific bacterium-antibiotic combination (Table 4). Generally, high levels of resistance of *E. coli* and *Salmonella spp* to all classes of antibiotics tested was observed. The pooled resistance rate of *E. coli* was lower with Gentamicin: 25.4% [(95% CI 10.1.0–91.7%)] compared to Trimethoprim/Sulfamethoxazole: 83.3% [(95% CI (51.3–96.0%)].

**Table 4:** Pooled prevalence of antibiotic resistance of *E. coli* and *Salmonella spp*-based on a meta-analysis of animal studies

Bacteria reported in studies	Antimicrobial agent	Number of studies	Pooled prevalence of AMR (95% CI)
*E. coli*	Aminoglycosides		
	Gentamicin	3	38.7 (7.8–82.4)
	Sulfonamides & Trimethoprim		
	Trimethoprim/Sulfamethoxazole	4	83.3 (51.3–96.0)
*Salmonella spp*	Beta-lactams		
	Amoxicillin	4	56.7 (8.0–95.2)
	Amoxicillin/Clavulanic acid	3	25.4 (10.1–91.7)
	Tetracyclines		
	Tetracycline	3	85.5 (49.9–97.7)
	Doxycycline	3	68.2 (57.2–75.5)

*AMR* Antimicrobial Resistance

**Fig. 8:**
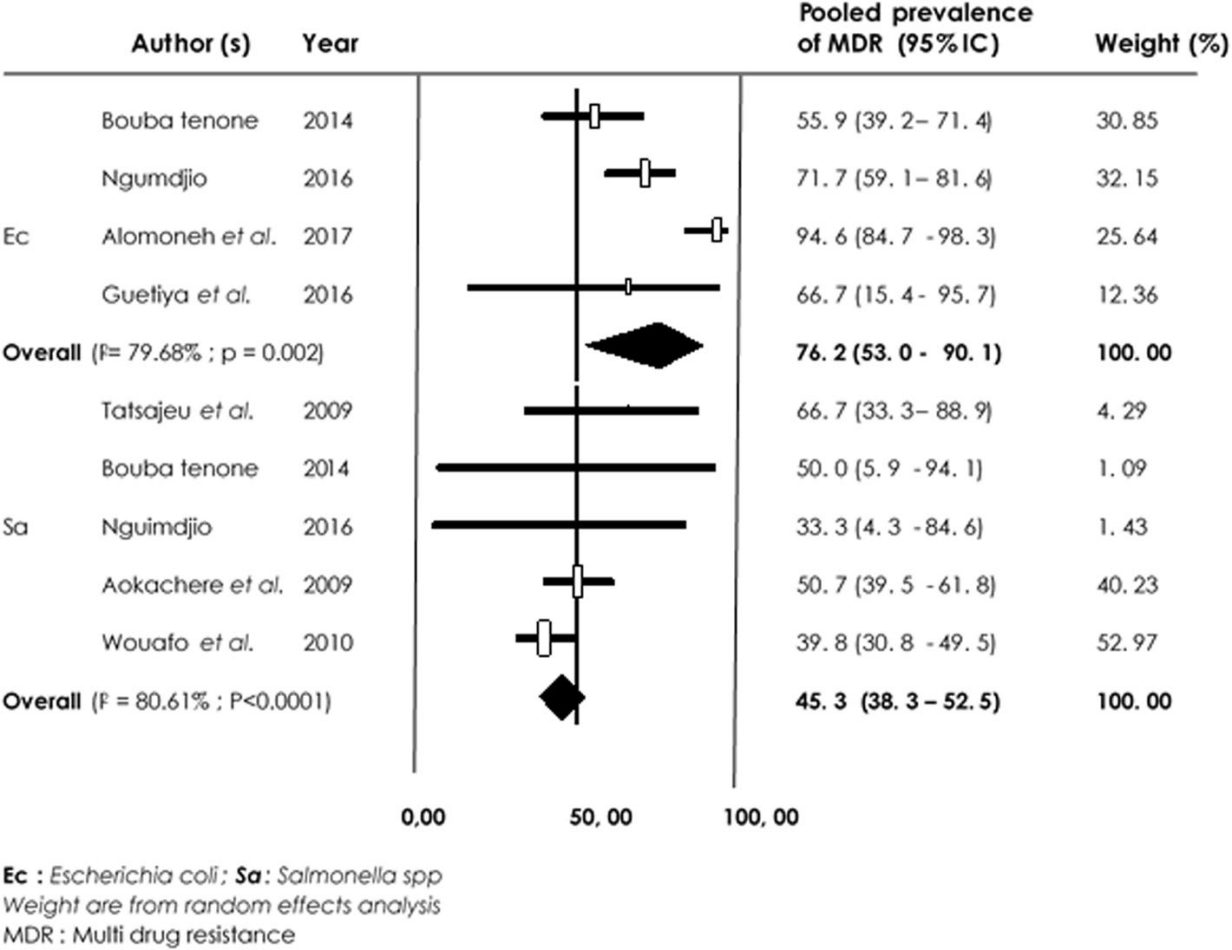
Forest plot of pooled prevalence of *E. coli* and *Salmonella spp*-multidrug resistance in animal

With regards to *Salmonella spp* from animal studies, Amoxicillin/Clavulanic acid was more susceptible compared to Tetracycline. Overall, MDR recorded for *E. coli* was 76.2% [95% CI (53.0–90.1%), I^2^ = 79.68% *p* = 0.002] and for *Salmonella spp* 45.3% [95% CI (38.3–52.5%), I^2^ = 80.61%; *p* < 0.0001] (Fig. 8).

### Antibiotic resistance rates in the environment

Out of 12 studies reporting antibiotic resistance in the environment, 9 were included in meta-analysis. Resistance rates were pooled if at least three studies reported on a specific bacterium-antibiotic combination (Table 5). Generally, a higher level of resistance of *Vibrio cholerae*, *Bacillus spp* and *Staphylococcus spp* to all classes of antibiotics tested was observed.

**Table 5:** Pooled prevalence of antibiotic resistance of *Bacillus spp, Staphylococcus spp* and *Vibrio cholerae*-based on the meta-analysis of environmental studies

Bacteria reported in studies	Antimicrobial agents	Number of studies	Pooled prevalence of AMR (95% CI)
*Bacillus spp*	Beta-lactams		
	Amoxicillin	4	83.8 (59.5–94.8)
	Amoxicillin+Clavulanic acid	3	65.9 (48.3–80.0)
	Penicillin	4	64.8 (34.2–86.7)
	Oxacillin	3	81.1 (64.8–90.9)
	Ceftazidime	3	53.4 (30.4–75.1)
	Cefuroxime	3	70.1 (50.6–84.2)
	Cefoxitine	3	88.7 (74.5–95.5)
	Ceftriaxone	3	61.7 (29.9–85.9)
	Aminoglycosides		
	Gentamicin	3	14.0 (6.6–27.1)
	Quinolones		
	Nalidixic acid	3	45.2 (20.8–72.3)
	Ciprofloxacin	3	28.1 (23.9–32.8)
	Nitrofuranes		
	Nitrofurantoin	3	60.4 (48.5–71.2)
	Sulfonamides & Trimethoprim		
	Co-trimoxazole	3	61.6 (38.1–80.7)
*Staphylococcus spp*	Beta-lactams		
	Amoxicillin	4	67.2 (31.1–90.3)
	Amoxicillin+Clavulanic acid	3	59.6 (47.5–70.6)
	Penicillin	5	78.3 (54.3–91.9)
	Oxacillin	3	89.3 (84.2–92.9)
	Ceftazidime	3	69.5 (52.0–82.7)
	Cefoxitine	3	74.7 (62.3–84.1)
	Ceftriaxone	3	83.8 (68.4–92.5)
	Quinolones		
	Nalidixic acid	3	89.1 (83.7–92.8)
	Ciprofloxacin	4	18.2 (5.9–44.3)
	Nitrofuranes		
	Nitrofurantoin	3	44.7 (37.9–51.7)
	Sulfonamides & Trimethoprim		
	Co-trimoxazole	3	62.1 (32.4–84.9)
*Vibrio cholerae*	Beta-lactams		
	Ampicillin	4	53.6 (47.3–59.8)
	Phenicols		
	Chloramphenicol	4	19.1 (2.4–69.0)
	Tetracyclines		
	Tetracycline	4	41.60 (14.6–74.9)

*AMR* Antimicrobial resistance

*Vibrio cholerae* recorded a lower rate of resistance to Chloramphenicol: 19.1% [(95% CI (2.4–69.0%)] compared to 53.6% [(95% CI (47.3–59.8%)] with Ampicillin. Pooled estimate of antimicrobial resistance prevalence of *Bacillus spp* was lower for Gentamicin: 14.0% [(95% CI (6.6–27.1%)] and Ciprofloxacin: 28.1% [(95% CI (23.9–32.8%)] compared to the third-generation cephalosporin Cefoxitine: 88.7% [(95% CI (74.5–95.5%)].

**Fig. 9:**
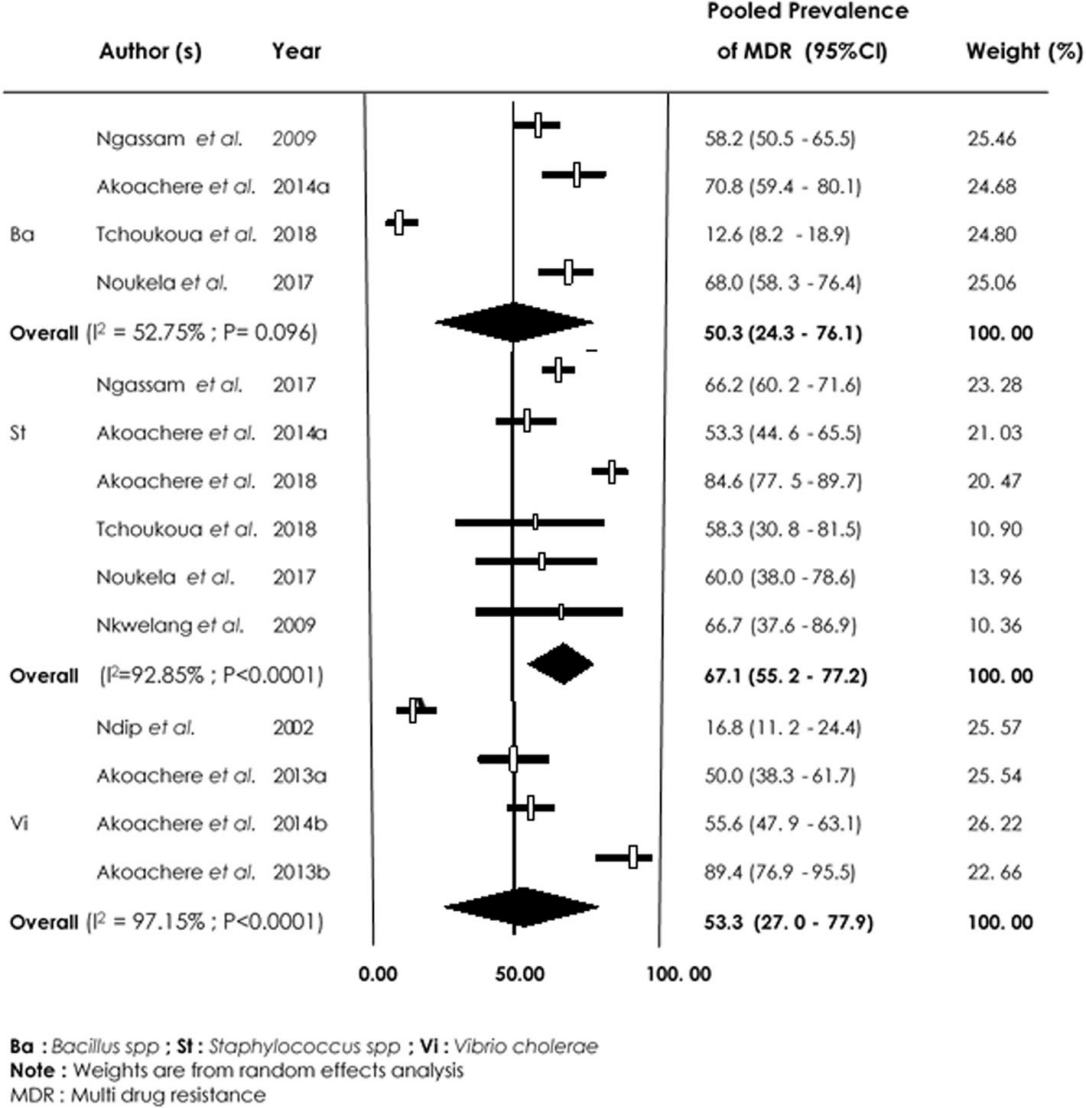
Forest plot of pooled prevalence of multidrug resistance in *Bacillus spp*, *Vibrio cholerae* and *Staphylococcus spp*- from the environment

Amongst isolated *Staphylococcus spp,* pooled estimate of prevalence rate was high in all molecule regularly used in hospital settings. Multidrug resistance (Fig. 9) of *Bacillus spp*, *Vibrio cholerae* and *Staphylococcus spp* of 50.3% [(95% CI (24.3–76.1%); I^2^ = 52.75%; *p* = 0.096], 53.3% [(95% CI (27.0–77.9%) I^2^ = 97.15%, *p* < 0.0001] and 67.1% [(95% CI (55.2–77.2%) I^2^ = 92.85%, *p* = 0.0001)] respectively, were recorded.

## Discussion

Antimicrobial resistance is a complex issue of global health concern. AMR is on the increase and threatens the effective management of most bacterial diseases worldwide [5, 84]. However, decreased death and morbidity rates associated with the use of antibiotics based on empirical guidelines are threatened by the development of AMR. Routine susceptibility checks of pathogens as essential keys geared towards strategies against the global AMR crisis have been previously described [10]. The One Health approach based on linking human health and nutrition with animal and environmental health is an improved multi-disciplinary action across these sectors against AMR. The current report reviewed 66 published data of resistance to commonly used antimicrobials in Cameroon. Overall, the review revealed that the level of resistance of bacteria isolated in hospital settings, animal and environment is high for typical empirical antibiotic treatment strategies. Common Enterobacteriaceae pathogens isolated in hospital settings such as *E. coli*, *Klebsiella* spp., *Enterobacter spp* and *Proteus spp* showed high rates of AMR to ampicillin, amoxicillin, trimethoprim/Sulfamethoxazole, amoxicillin/ clavulanic acid, tetracycline, co-trimoxazole and nalidixic acid. The finding agrees with data obtained in Tanzania (resistance to co-trimoxazole (77.7%) and ampicillin (81.6%) [85] and East Africa (50–100% resistance to ampicillin and cotrimoxazole) [4].

This review highlights serious concerns relating to the use of ampicillin, amoxicillin and tetracycline as the antibiotics of choice for optimal therapy of common pathogens in Cameroon. Vital information on the burden of antibiotic resistance and concerns on diagnostic capacity and second-line treatment options in the country are also presented. The misuse of antibiotics such as inappropriate prescription of antibiotics, excessive use and auto-medication and insufficient hygienic practices in health care settings have been reported to contribute to rising levels of AMR [86, 87]. The moderate resistance level (42%) observed for *Staphylococcus spp* isolated from hospital settings to third generation cephalosporins, gentamicin and fluoroquinolones was due to the fact that these drugs were usually the last choice of medicine used. This suggests that the choice of antibiotic should be guided by a profile of antimicrobial susceptibility testing results of patients in Cameroon. Multidrug resistance (more than 10 antibiotics) of common bacteria such as *E. coli* (47.1%), *Klebsiella spp* (51.0%) and *Staphylococcus spp* (45.2%) were further evidence of high and widespread AMR in Cameroon. The review revealed that AMR is a growing health problem and major concerns among hospitalized persons and risk communities. Generally, antibiotic resistance is under-investigated and under-reported in the WHO African region including Cameroon [1, 5, 11] due to limited or unavailability of diagnostic tests and lack of microbiology technical resources. These have led to the inability to test clinical isolates for antimicrobial susceptibility as well as contributed to excessive use and misuse of antibiotics and the phenomenon of AMR. Antibiotic resistant strains may be easily transferred within the human-animal-environment interfaces due to high level of interactions. For example, resistant pathogens in food producing animals and environments can be passed easily to human during various interactions.

Pathogens isolated from food producing animals in the current review such as *Salmonella spp* and *E. coli* showed high resistance against ampicillin, amoxicillin, STX, tetracycline and doxycycline. This agrees with reported high antibiotic simple (up to 86%) and multidrug (73%) resistance rates in Africa [88]. Considering that these are typically empiric antibiotics used for strategic health management in human and animals, the preservation of these important antibiotics for human health cannot be overemphasized [89, 90]. The multidrug resistance observed in this study for *Salmonella spp* and *E. coli,* could be a consequence of overuse or misuse of antibiotics, failure to consult veterinarians before the antibiotherapy, absence of a clear regulation code on the use of antibiotics in animals [91, 92] in Cameroon. The widespread use of substandard drugs [93] and poor hygiene handling of food [94, 95] have been reported earlier in the country. The main route of transmission of drug-resistant strains from food producing animals to humans occur through contamination of edible animal products. Therefore, the misuse of veterinary drugs and misguided therapy in veterinary medicine could select resistant strains in food animals and cause considerable impact on human health [96].

The findings of this review revealed gaps in the surveillance and interdisciplinary sharing of data on the emergence of AMR in foodborne bacteria and their potential impact on both animal and human health in Cameroon. In this context, the One Health Approach for control AMR should include improved integrated surveillance of resistance in bacteria in food-producing animals in the food chain, food products, humans and the environment and prompt sharing of data [96].

Uncontrolled use and/or misuse of antimicrobials (use without prior laboratory susceptibility testing) in agriculture has been linked to increase in the rates of drug resistance [5, 84] since contaminating bacteria usually tend to be treated with dosage rates lower than required for the treatment or exposed to an ineffective drug. In this review, *Staphylococcus spp*, *Bacillus spp* and *Vibrio cholerae* isolated from the environment (hospital surfaces and devices, water and abattoirs drain) were highly resistant to the first line antibiotics used in human and animals and showed high multidrug resistance rates. Poor hygiene of hospital surfaces and inadequate management of expired drugs could be responsible for the high AMR rates. Easy transmission of resistant bacteria from the environment to humans and animals also play a role in the highly resistant *Staphylococcus spp* reported in human studies and most of these inappropriately used antimicrobials often end up in wastewater in low and middle-income countries including Cameroon as previously reported [97]. Drug-resistant pathogens have higher chances of surviving and emerging when they are found in sewage. Also, antibiotic-resistant bacteria in the environment may be due to the dynamism and acquisition of antibiotic resistance genes that can be transferred horizontally from one bacteria to another [3]. This could explain high relationship between resistance in the environmental studies and overuse and uncontrolled use of antimicrobials in human and animal medicine since part of the drugs are excreted or secreted in the environment [3].

The current review also highlights lack of published AMR data on human, animal and environmental health in three, six and seven regions in Cameroon, respectively. Bridging the knowledge gaps and enhancing AMR surveillance in Cameroon to reduce the threat of AMR on public health cannot be overemphasised. As part of the global One Health Strategic plan against public threats and enhance the Global Antimicrobial Resistance Surveillance System [20], Cameroon has instituted a National Action Plan to fight against Antimicrobial Resistance (NAP-RAM 2018–2020) [98] to reduce the burden of AMR in the country. The plan is multisectoral involving seven key ministerial departments and technical partners and has six major objectives: (1) to improve awareness and understanding of antimicrobial resistance through effective communication, education and training; (2) to strengthen knowledge and evidence through monitoring and research; (3) to reduce the incidence of infections by applying effective sanitation, hygiene and infection control measures; (4) to optimize the use of antimicrobials in human, animal and plant health; (5) to develop economic arguments for sustainable investments and the production of new medicines, diagnostic tools, vaccines and other interventions for the fight against AMR; (6) to improve governance in the sector through strengthening of standardization, regulation and accountability. Effective implementation of the actual national action plan according to the “One Health” concept could be a reliable effort to reduce the burden of AMR at the national level. However, certain barriers for implementation exist including as poverty, remote nature of many rural zones and risk of emerging diseases. In addition, the lack of experts trained in ‘One Health Approach’ and the inadequacy of curriculum of educational programs regarding antimicrobial prescription, use and AMR could constitute other limitations to the application of this current plan.

This review does not only give an estimate of the level of AMR in Cameroon but revealed large knowledge gaps. Short comings were related to the representativeness of data which focused on AMR reports which did not support evaluation since absence of resistance cases were not routinely declared. Existing data were concentrated on four regions of the country. Also, very few reports from animal and environment were available. A further limitation is combining AMR results from different patient groups across different regions of the country to compare. The high-level resistance rates observed could be due to the approach used. Nonetheless, the review revealed the general trend and developments of AMR. Finally, for the purpose of this review, resistance data gotten using laboratory methodologies were combined. Variation in AMR methodology has minimal effect on the quality of final results and many studies used the disk diffusion method and CLSI guidelines [1]. Intense therapeutic studies will highlight further knowledge on the clinical impact of AMR and disadvantages of strategies that rely on empirical use of broader-spectrum drugs. The emergence of AMR in Cameroon is a real threat that cannot be ignored.

## Conclusion

The present review highlights the high prevalence of resistance to commonly used antibiotics in humans, animals and the environment in Cameroon. Multi-drug resistance was observed to be a real and rising threat. All bacteria isolated showed resistance to more than one drug of choice in different or similar drug lines. In the Cameroon context where infectious diseases are highly endemic, there is insufficient data from interventional studies in the struggle to contain AMR. Given that there is dearth of data on hospital, community-acquired infections, animal infections and prevalence of AMR in Cameroon, systemic investigations and surveillance cannot be overemphasized. Long-lasting measures encouraging the implementation of antimicrobial stewardship in Cameroon such as pharmacy, laboratory quality control, full microbiology investigations and the development and distribution of standard antibiograms nationwide should be reinforced. Public health education on prescription, sensitization of users and elaboration of stringent drug regulations and fight against counterfeit drugs are essential to reduce AMR in Cameroon. The national action plan to reduce the burden of AMR can succeed only through continued data sharing as well as global collaboration, harmonization, and coordination between all partners involved in the implementation of AMR surveillance.

## Data Availability

Not applicable.

## Abbreviations

AMR: Antimicrobial Resistance
CI: Confidence Interval
GLASS: Global Antimicrobial Resistance Surveillance System
MDR: Multidrug Resistance
PRISMA: Preferred Reporting Items for Systematic Reviews and Meta-Analyses
WHO: World Health Organization
